# Erythema nodosum leads to the diagnosis of pulmonary tuberculosis

**DOI:** 10.11604/pamj.2014.18.291.5170

**Published:** 2014-08-14

**Authors:** Theocharis Koufakis, Ioannis Gabranis

**Affiliations:** 1 Department of Internal Medicine, General Hospital of Larissa, Larissa, Greece

**Keywords:** Erythema nodosum, Tuberculosis, Hilar lymphadenopathy

## Abstract

Erythema nodosum is a panniculitis which may have various causes, such as drugs, infections, sarcoidosis, inflammatory bowel disease, tuberculosis or can be idiopathic. We here report a case of a womandiagnosed with pulmonary tuberculosis whose first symptom was erythema nodosum. A thorough clinical and laboratory investigation of the patient presenting with erythema nodosum is always required, in order to assess a possible systemic, underlying condition.

## Introduction

Erythema nodosum is a panniculitis, characterized by red, tender nodules usually located on the extensor surfaces of the legs. It may have various causes, such as drugs, infections, sarcoidosis, inflammatory bowel disease, tuberculosis or can be idiopathic [1]. In up to 50% of cases, the underlying etiology remains unclear [2].

## Patient and observation

**Figure 1:**
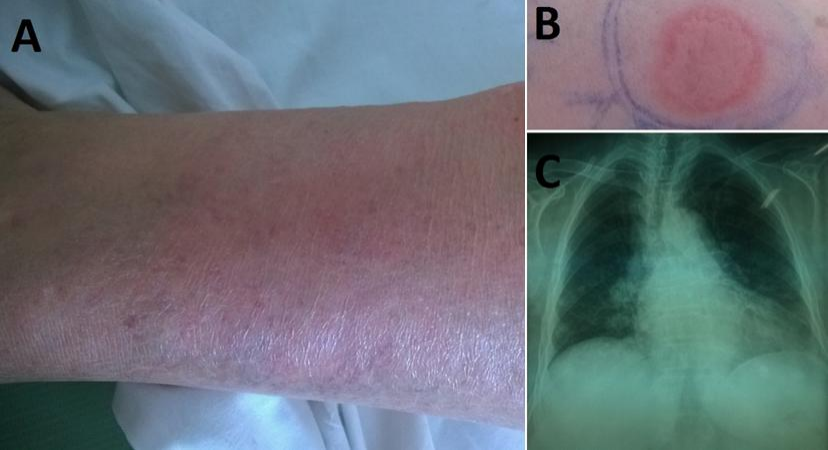
A) Erythema nodosun on the patient's left shin; B) Positive Mantoux test; C) Chest X-Ray demonstrating hilar lymphadenopathy, mainly at the right side

We here report a case of a 78 year old woman, with free medical history, who presented to the Emergency Department of our Hospital with erythema nodosum on her both shins (Figure 1A). She also mentioned low grade fever since 15 days and a positive family history of tuberculosis (daughter). Her chest X-Ray revealed bilateralhilar lymphadenopathy, mainly at the right side (Figure 1C). Further evaluation with High Resolution chest Computed Tomography confirmed the lymphadenopathy and demonstrated ground-glass opacities. The Mantoux skin test was strongly positive (22 mm) (Figure 1B). Anti-TB treatment was started, based on the positive Mantoux test, the compatible imaging findings and mainly, the history of close and direct exposure. Sputum cultures proved to be positive for Mycobacterium tuberculosis. Patient's response to treatment was impressive and in her follow up visits, she remained free of symptoms and a define improvement of her imaging findings was observed.

## Discussion

The commonest causes of erythema nodosum reported in the literature, are streptococcal infection in children and streptococcal infection and sarcoidosis in adults [3]. Its course is, in most cases, benign and self-limited. However, therapeutic options include, among others, NSAIDS, corticosteroids antimicrobial agents and colchicine.

## Conclusion

In conclusion, a thorough clinical and laboratory investigation of the patient presenting with erythema nodosum is always required, in order to assess a possible systemic, underlying condition.
